# Effects of melatonin implantation on carcass characteristics, meat quality and tissue levels of melatonin and prolactin in Inner Mongolian cashmere goats

**DOI:** 10.1186/s40104-019-0377-y

**Published:** 2019-09-02

**Authors:** Tao Duan, Ziyuan Wu, Huan Zhang, Ying Liu, Yan Li, Wei Zhang

**Affiliations:** 1 State Key Laboratory of Animal Nutrition, College of Animal Science and Technology, China Agricultural University, Beijing, 100193 People’s Republic of China; 2 Beijing Sunlon Livestock Development Company, Beijing, 100076 People’s Republic of China; 3 College of Animal Science, Zhejiang University, Hangzhou, 310085 People’s Republic of China

**Keywords:** Carcass characteristics, Cashmere goat, Meat quality, Melatonin

## Abstract

**Background:**

Implantation of goats with melatonin can induce cashmere growth and significantly increase cashmere production performance. However, the impact of melatonin implantation on the carcass characteristics, meat quality and related hormone levels in muscle and viscera of cashmere goats has not been studied. This experiment was conducted to determine the effects of melatonin implantation of cashmere goats during the non-growing period on meat quality and related hormone levels in the tissues. It aimed to provide a theoretical basis for the practical application of melatonin in cashmere goat production systems.

**Results:**

Melatonin implantation (2 mg/kg live weight) had no influence (*P* > 0.05) on daily weight gain, carcass weight, dressing percentage, loin muscle area, or the pH, moisture level, crude fat (except for *Gluteus* muscle) and amino acid content of muscles of cashmere goats. After implantation for 1 month, shear force of *Longissimus dorsi* and water loss rate of *Longissimus dorsi* and *Biceps femoris* of cashmere goats were increased (*P* < 0.05), whereas the cooking yield of *Gluteus* muscle was reduced (*P* < 0.05). The melatonin treatment decreased (*P* < 0.05) muscle crude protein, *Gluteus* muscle crude fat and ∑*n*-3PUFA content and decreased (*P* < 0.05) ∑*n*-6PUFA content. However, after 2 months of implantation most of these effects had resolved. Melatonin implantation had no effect (*P* > 0.05) on the melatonin or prolactin contents of kidney, heart, spleen, liver, *Longissimus dorsi*, *Biceps femoris* and *Gluteus* muscles. Melatonin content of lung tissue was lowered (*P* < 0.05) and that of prolactin was elevated (*P* < 0.05) by the melatonin implantation.

**Conclusion:**

This study has shown little impact of melatonin implantation of cashmere goats on carcass quality. A few meat quality indices i.e., shear force, water loss rate, ∑*n*-3PUFA, ∑*n*-6PUFA, and crude protein content of *Longissimus dorsi*; water loss rate, cooking yield and crude protein content of *Biceps femoris*; ether extract, crude protein content of *Gluteus*; were affected briefly (at 1 month of implantation) but these effects were not evident after 2 months of implantation. There was little effect of the melatonin treatments on tissue levels of melatonin or prolactin except in lung.

## Background

The Inner Mongolian cashmere goat is a major breed of goat in China. It produces cashmere with an average fiber diameter of less than 15 μm and a fiber length ranging from 4 to 8 cm [1]. China is globally the largest producer of cashmere, producing about 17.85 million kg cashmere in 2017, with 44.96% of this from the Inner Mongolia Autonomous Region [2]. China is the primary source of the finest quality cashmere (14–15 μm) which is used in knitted garments [3]. Growth of the cashmere fleece is seasonal and, thus, is linked to changes in the natural daily photoperiod [4].

It is well established that melatonin is a pivotal intermediary between the environmental photoperiod and the growth of cashmere fibers, the latter being directly influenced by the circulating levels of melatonin [5, 6]. The effects of implantation of goats with exogenous melatonin on the production of cashmere have been studied during its peak growing period [7], during the slow-growing period [8–10] and during the non-growing period [6, 11]. Almost three decades ago Welch et al. [12] reported that melatonin implantation of goats during the non-growing period promoted cashmere growth and extended its growth phase. More recently, Duan et al. [11] and Chu et al. [13] have developed use of melatonin implants (2 mg/kg live weight) on two occasions during the non-growing period (late April and June) as a practical method for enhancing cashmere production in seasonal breeds such as Inner Mongolian cashmere goats.

In addition to cashmere, meat is an important product in the cashmere goat industry and is a major source of income for farmers and herdsmen. However, consumer acceptance of meat products is greatly affected by their quality. Consumer preferences for meat tend to be determined by its physical characteristics, whereas its nutritional quality is more closely related to the chemical composition [14]. Treatment of finishing pigs with feeding melatonin has a negative effect on muscle pH and water loss rate, but this may be a degree of dose dependence [15], injection treatment can also affect pork quality [16]. To the best of our knowledge, there are no published reports on the effects of melatonin implantation on the carcass characteristics and meat quality of cashmere goats. Hence, the primary purpose of the present study was to investigate the effects of melatonin implantation of cashmere goats on live weight gain, carcass characteristics and meat quality, and on melatonin and prolactin levels in tissues. The aim was to provide a theoretical basis for the practical application of melatonin implantation technology in cashmere goat production systems.

## Methods

### Animals and experimental design

This experiment was performed at the YiWei White Cashmere Goat Farm located in the Inner Mongolia Autonomous Region of China (39°06′N, 107°59′E, at an altitude of 1,500 m) from 15^th^ May to 15^th^ July, 2014. All procedures used in this study were approved by the Animal Care and Use Committee of China Agricultural University (Beijing, China).

Eighteen 1-year-old well-conditioned Inner Mongolian cashmere wethers with an initial body weight of 25.67 ± 0.62 kg were randomly assigned to three groups (*n* = 6), including a control group (Group C), a group which melatonin microcapsules (Beijng Kangtai Biological Technology Company, Beijing, China) were implanted subcutaneously in the base of one ear on 15^th^ May (Group MM) and a group implanted likewise on 15^th^ June (Group MJ). The microcapsules were comprised of medical grade silicone elastomer impregnated with melatonin that could be slowly released for at least 2 months. The melatonin dosage used here (2 mg/kg live weight) was based on that of Duan et al. [11, 17]. Group C goats received no treatment. At the end of the study period (15^th^ July) jugular blood samples were collected by venipuncture and the goats were slaughtered for subsequent sampling of various muscles and other tissues.

During the experimental period, all goats were maintained under natural photoperiodic conditions. Feeding and management of the goats was typical of that for other cashmere goats at the farm where goats are kept permanently on a desert pasture with occasional supplementary feeding. Goats were provided with grazing and supplementary feeding management from January to June, with 0.275 kg/d concentrate (70% corn and 30% condensed feed purchased from Baotou Jiuzhoudadi Biotech Company, Baotou, China) per goat provided in January, which was gradually increased to 0.4 kg/d in April and subsequently increased to 0.55 kg/d in May and June. Goats were grazed on natural pasture without supplementary feeding from July to December. In this region, the primary vegetation includes *Caragana stenophylla* Pojark, *Caragana rorsninskii* Kom, *Agropyron cristatum* Gaertn*, Agropyron cristatum* Schult*, Allium polyrhizum* Turcz*, Artemisia frigida* Willd*, Artemisia ordosica* Krasch*, Stipa breviflora* Griseb *and Haloxylon ammodendron* Bunge*,* some of which is grazed only by goats [18].

### Slaughter procedure and sample collection

The goats were fasted for 24 h with free access to water until 2 h before slaughter. They were weighed and then slaughtered by the standard Halal procedure whilst taking care to avoid undue stress to the animals. Samples (approximately 300 g) were dissected from the *Longissimus dorsi* muscle, between the 12^th^ thoracic and 5^th^ lumbar vertebrae, from the *Biceps femoris* muscle and from the *Gluteus* muscle, all on the left half of the carcass. The muscle samples were placed into polyethylene bags and stored at − 20 °C prior to determining routine meat quality parameters, nutrient content and contents of melatonin and prolactin. Approximately 50 g samples were dissected from the kidney, heart, spleen, liver and lung, then frozen at − 20 °C prior to determining contents of melatonin and prolactin.

### Determination of carcass characteristics

The dressed carcass, which comprised the body after removing the skin, head, fore feet, hind feet and viscera, was weighed approximately 30 min post mortem. Dressing percentage was calculated using the following formula: dressing percentage (%) = (dressed carcass weight / antemortem live weight) × 100. Sulfuric acid paper was used to gain an imprint of a cross section of the *Longissimus dorsi* muscle at the junction of the last thoracic spine and the first lumbar spine of the left side of each carcass and the area of the imprint was determined by planimetry.

### Assessment of meat quality

Meat quality was assessed by determining muscle pH, tenderness and color, and by measuring water loss rate and cooking yield. The pH at 45 min post mortem (pH_45_) was measured using a pH meter (TESTO 205, Testo Limited, Alton, Hampshire, UK) by inserting the electrode into three different points of the *Longissimus dorsi*, *Biceps femoris* and *Gluteus* muscles which had been trimmed into strips (5 cm × 1 cm × 0.5 cm) without fascia, epimysium or fat. Up to 3 muscle strips (2.5 cm × 3 cm × 5 cm) were removed as close to the longitudinal orientation of the muscle fibers as possible. After sealing, the meat strips were heated in a 75 °C water bath for 45 min, cooled to room temperature, and the surface moisture was wiped off with a filter paper. From each sample, 3 replicate strips (1 cm × 1 cm × 2 cm) were cut parallel to the direction of the muscle fibers and each strip was sheared in the center perpendicular to the longitudinal direction of the fibers [19]. Tenderness of these muscles was determined by measuring shear force in triplicate with a tenderness apparatus (C-LM3, Northeast Agricultural University, College of Engineering, Harbin, China) fitted with a Warner-Bratzler blade and the blade speed was set at 1 mm/s in the process of shearing. The measurement accuracy is 1%. Muscle color was determined with an automated colorimeter (TC-P2A, illuminant D65, 10°standard observer, Aoike Optoelectronic Instrument Co., Ltd., Beijing, China) using a standard whiteboard as a comparative reference. Hunter lightness (L*), redness (a*) and yellowness (b*) values were measured in triplicate. Water loss rate was determined by the pressure method. For this, three 1 cm thick samples of muscle were cut with a 2.532-cm diameter circular sampler (about 5 cm^2^ area) and weighed immediately on an analytical balance to give the pre-pressure weight (*W*_1_). The samples were placed on a platform with 18 layers of qualitative medium-speed filter paper above and below, were applied 35 kg pressures for 5 min, and were then re-weighed to get the post-pressure weight (*W*_2_). Water loss rate was calculated using the following formula: water loss rate (%) = [(*W*_1_−*W*_2_)/*W*_1_] × 100. For cooking yield, muscle samples (about 50 g) were weighed (*W*_1_) then placed in flat dishes and steamed for 45 min, cooled to room temperature, blotted dry and reweighed (*W*_2_). Cooking yield was calculated using the following formula: cooking yield (%) = (*W*_2_/*W*_1_) × 100. For all of these meat quality measurements, the mean of the triplicates was recorded as the final value for analysis.

### Determination of chemical composition of muscles

Chemical composition of muscles was analyzed in duplicate according to AOAC methods (1997) [20]. Moisture content was determined gravimetrically by oven drying and crude protein and ether extract contents were measured using Kjeldahl and Soxhlet extraction methods, respectively [21].

### Determination of amino acid and fatty acid content of *Longissimus dorsi*

The amino acid content of 100 mg samples of *Longissimus dorsi* was determined with an automated dedicated amino acid analyzer (Hitachi 835, Japan) following acid hydrolysis (10 mL of 6 mol/L HCl for 22 h at 110 °C in nitrogen gassed atmosphere), with vacuum drying and volume equilibration using 0.02 mol/L HCl prior to detection. Fatty acid content was determined by gas chromatography (HP6890, Hewlett-Packard, Avondale, PA, USA).

### Determination of melatonin and prolactin levels

Melatonin and prolactin concentrations of muscle and tissues were determined by radioimmunoassays using a gamma counter (r-911, University of Science and Technology of China, Industry Corporation, Hefei, China) with assay services supplied by Beijing Sino-uk Institute of Biological Technology. Briefly, a portion (0.3 g~ 0.5 g) of meat or tissues without tissue fluid and blood on the surface was homogenized with 1 mL normal saline which containing 20 μL of acetic acid (0.05 mol/L), the homogenate was centrifuged at 3,000 r/min for 20 min and the supernatant was taken. A total of 0.5 mL normal saline which containing 10 μL of acetic acid (0.05 mol/L) was added to the precipitate, then centrifuged and the supernatant was taken. After that the two supernatants was mixed and the pH value was adjusted to 7.4 using 0.05 mol/L NaOH. Melatonin concentrations were analyzed using a commercial radioimmunoassay kit (HY-10177, Sino-uk Institute of Biological Technology, Beijing, China) according to the manufacturer’s instruction. The intra- and inter-assay CV were 8.3% and 14%, respectively. The sensitivity of the assay was 1.0 pg/mL. For detection of prolactin, the samples were homogenized with 1 mL of absolute ethanol, and the supernatant was taken after centrifugation. The precipitate was further extracted by adding absolute ethanol and the supernatant was taken after centrifugation. Then combine the two supernatants to dectect the prolactin using a ^125^I-prolactin radioimmunoassay kit (HY-10026, Sino-uk Institute of Biological Technology, Beijing, China) according to the manufacturer’s instruction. The intra- and inter-assay CV were 5% and 10%, respectively. The sensitivity of the assay was 0.1 ng/mL.

### Statistical analysis

Data were analyzed as a completely randomized design using a one-way analysis of variance (SPSS 20.0, International Business Machines, Armonk, NY, USA). Duncan’s method for multiple comparisons was used if treatment effects were detected. The level of statistical significance was set at *P* < 0.05. The data are expressed as the mean ± SEM.

## Results

### Carcass characteristics

There were no differences (*P >* 0.05) in mean carcass weight, dressing percentage or cross sectional area of the *Longissimus dorsi* muscle among the control and melatonin implanted groups of these cashmere goats (Table 1).

**Table 1:** Effects of melatonin treatment on carcass characteristics of Inner Mongolian cashmere goats^1^

Items	Group C	Group MM	Group MJ
Carcass weight, kg	11.4 ± 0.6	12.0 ± 0.3	11.8 ± 0.1
Dressing percentage, %	39.4 ± 1.7	41.7 ± 0.7	41.8 ± 1.9
Muscle area^2^, cm^2^	11.3 ± 0.2	11.0 ± 0.7	10.9 ± 0.5

^1^ Values are means of six replicates per treatment. Group C = controls, Group MM = melatonin implanted in May, Group MJ = melatonin implanted in June

^2^ Cross sectional area of *Longissimus dorsi* muscle

### Meat quality and chemical composition of muscle

Generally, there were few effects of the melatonin treatment on meat quality indices (Table 2). One month of melatonin implantation (Group MJ) increased (*P* < 0.05) mean shear force and water loss rate of *Longissimus dorsi* muscles and reduced (*P* < 0.05) cooking yield and ether extract content of *Gluteus* muscles in comparison with the corresponding muscles from goats in Groups MM and C (Table 2). The cooking yield of *Gluteus* muscles remained significantly (*P* < 0.05) reduced at 1 month (Group MJ) (Table 2). Melatonin treatment for 1 month increased (*P* < 0.05) water loss rate of *Biceps femoris* muscles of Group MJ goats in comparison with those of controls (Group C) (Table 2). The 1 month of melatonin implantation (Group MJ) reduced (*P* < 0.05) muscle crude protein content below that of controls and reduced (*P* < 0.05) crude protein content of *Longissimus dorsi* and *Biceps femoris* muscles below that of goats implanted with melatonin for 2 months (Group MM) (Table 2).

**Table 2:** Effects of melatonin treatment on meat quality indices of Inner Mongolian cashmere goats^1^

Items		Muscle	Group C	Group MM	Group MJ
pH_45_^2^		*Longissimus dorsi*	6.04 ± 0.12	6.06 ± 0.23	6.03 ± 0.11
		*Biceps femoris*	6.47 ± 0.17	6.34 ± 0.10	5.83 ± 0.49
		*Gluteus*	6.54 ± 0.10	6.40 ± 0.10	6.36 ± 0.10
Meat color^3^	L*	*Longissimus dorsi*	37.05 ± 1.06	32.30 ± 0.96	34.42 ± 1.71
		*Biceps femoris*	36.58 ± 2.05	33.35 ± 1.20	35.70 ± 1.69
		*Gluteus*	33.97 ± 1.16	35.47 ± 3.19	33.65 ± 1.71
	a*	*Longissimus dorsi*	14.31 ± 0.84	15.29 ± 1.23	15.59 ± 0.82
		*Biceps femoris*	16.75 ± 0.97	17.28 ± 1.51	18.78 ± 0.73
		*Gluteus*	17.46 ± 1.29	15.94 ± 2.03	18.70 ± 1.23
	b*	*Longissimus dorsi*	4.78 ± 0.44	4.61 ± 0.62	3.99 ± 0.34
		*Biceps femoris*	5.62 ± 0.34	5.02 ± 1.14	3.95 ± 0.70
		*Gluteus*	4.82 ± 0.65	5.08 ± 0.78	5.22 ± 0.55
Shear force, kgf^4^		*Longissimus dorsi*	5.10 ± 0.05^a^	5.32 ± 0.10^a^	5.50 ± 0.16^b^
		*Biceps femoris*	7.24 ± 0.07	7.42 ± 0.12	7.54 ± 0.13
		*Gluteus*	5.51 ± 0.11	5.54 ± 0.11	5.64 ± 0.07
Water loss rate, %		*Longissimus dorsi*	23.5 ± 0.39^a^	24.38 ± 0.44^a^	26.82 ± 0.36^b^
		*Biceps femoris*	19.27 ± 0.60^a^	20.98 ± 0.28^ab^	21.79 ± 0.16^b^
		*Gluteus*	24.98 ± 0.50	25.15 ± 0.61	26.29 ± 0.22
Cooking yield, %		*Longissimus dorsi*	67.21 ± 0.32	67.53 ± 0.37	67.27 ± 0.48
		*Biceps femoris*	63.01 ± 0.51	63.25 ± 0.44	62.39 ± 0.35
		*Gluteus*	65.51 ± 0.38^a^	65.39 ± 0.26^a^	64.11 ± 0.27^b^
Moisture content, %		*Longissimus dorsi*	72.90 ± 0.40	72.73 ± 0.33	72.77 ± 0.49
		*Biceps femoris*	71.95 ± 0.38	72.60 ± 0.39	72.70 ± 0.43
		*Gluteus*	72.39 ± 0.37	72.43 ± 0.36	72.60 ± 0.44
CP^5^, %		*Longissimus dorsi*	21.42 ± 0.11^a^	21.22 ± 0.24^a^	19.86 ± 0.36^b^
		*Biceps femoris*	21.45 ± 0.18^a^	21.24 ± 0.21^a^	20.32 ± 0.39^b^
		*Gluteus*	21.37 ± 0.19^a^	20.88 ± 0.39^ab^	20.02 ± 0.31^b^
EE^6^, %		*Longissimus dorsi*	11.06 ± 0.32	10.30 ± 0.37	9.95 ± 0.36
		*Biceps femoris*	11.28 ± 0.43	11.27 ± 0.25	10.28 ± 0.36
		*Gluteus*	11.45 ± 0.34^a^	10.98 ± 0.29^a^	9.80 ± 0.25^b^

^1^ Values are means of six replicates per treatment. Group C = controls, Group MM = melatonin implanted in May, Group MJ = melatonin implanted in June

^2^ pH_45_ represents pH value for muscles 45 min after slaughter

^3^ L* = lightness, a* = redness, b* = yellowness

^4^ kgf = kilogram-force. 1 kgf = 9.80665 N

^5^ CP = crude protein content

^6^ EE = ether extract content

^a, b^ Mean in the same row with different superscripts are significantly different (*P* < 0.05)

### Amino acids and fatty acids in *Longissimus dorsi* muscle

The content of various amino acids in *Longissimus dorsi* muscles of these goats was not affected (*P* > 0.05) by melatonin implantation (Table 3). Melatonin implantation did not significantly influence either 7 kinds of essential amino acids, 10 kinds of non-essential acids or total-amino acids, essential amino acid content and the ratio of essential amino acid to total amino acids (*P* > 0.05).

**Table 3:** Effects of melatonin treatment on amino acid composition of *Longissimus dorsi* muscle in Inner Mongolian cashmere goats^1^

Items		Group C	Group MM	Group MJ
EAA^2^, mg/100 mg	Thr	3.63 ± 0.05	3.62 ± 0.06	3.60 ± 0.04
	Val	4.19 ± 0.06	4.17 ± 0.07	4.15 ± 0.05
	Met	2.28 ± 0.04	2.25 ± 0.03	2.26 ± 0.03
	Ile	4.03 ± 0.08	4.00 ± 0.07	4.01 ± 0.05
	Leu	6.85 ± 0.06	6.87 ± 0.14	6.93 ± 0.35
	Phe	4.17 ± 0.27	4.19 ± 0.28	4.41 ± 0.72
	Lys	7.76 ± 0.22	7.99 ± 0.22	8.47 ± 0.46
NAA^3^, mg/100 mg	Asp	7.48 ± 0.12	7.47 ± 0.12	7.40 ± 0.10
	Tyr	3.02 ± 0.25	3.06 ± 0.24	3.27 ± 0.68
	His	2.91 ± 0.16	3.15 ± 0.23	3.77 ± 0.65
	Arg	5.18 ± 0.07	5.17 ± 0.08	5.13 ± 0.07
	Ser	2.94 ± 0.03	2.95 ± 0.04	2.93 ± 0.04
	Glu	14.55 ± 0.20	14.55 ± 0.21	14.60 ± 0.67
	Pro	2.65 ± 0.08	2.71 ± 0.09	2.66 ± 0.08
	Gly	3.38 ± 0.05	3.46 ± 0.10	3.32 ± 0.08
	Ala	4.64 ± 0.06	4.66 ± 0.07	4.61 ± 0.06
	Cys	0.50 ± 0.03	0.54 ± 0.02	0.52 ± 0.02
TAA^4^, mg/100 mg		80.14 ± 0.54	80.89 ± 0.38	82.08 ± 0.47
EAA, mg/100 mg		32.90 ± 0.72	33.09 ± 0.68	33.85 ± 0.68
EAA/TAA, %		41.04 ± 0.16	40.94 ± 0.24	41.18 ± 0.30
EAA/NAA, %		69.62 ± 0.47	69.34 ± 0.70	70.03 ± 0.89

^1^ Values are means of six replicates per treatment. Amino acid content is based on freeze-dried condition. Group C = controls, Group MM = melatonin implanted in May, Group MJ = melatonin implanted in June

^2^ EAA = essential amino acids

^3^ NAA = non-essential amino acids

^4^ TAA = total-amino acids

^a, b^ Mean in the same row with different superscripts are significantly different (*P* < 0.05)

There was also minimal effect of melatonin implantation on the fatty acid composition of the *Longissimus dorsi* muscles of these goats (Table 4). Goats implanted with melatonin, especially those implanted in June (Group MJ, 1 month) had elevated (*P* < 0.05) proportions of C14:1, C15:1, C20:4(*n*-6) and ∑*n*-6PUFA fatty acids and lower (*P* < 0.05) proportions of C12:0, C18:2C, C18:3(*n*-3) and ∑*n*-3PUFA in *Longissimus dorsi* muscles (Table 4). The ratio of ∑*n*-6/∑*n*-3 in group MJ was significantly higher than in group C (*P* < 0.05).

**Table 4:** Effects of melatonin implantation on fatty acid content of *Longissimus dorsi* in Inner Mongolian cashmere goats^1^

Fatty acid, mg/g	Group C	Group MM	Group MJ
C10:0	0.07 ± 0.01	0.08 ± 0.01	0.07 ± 0.003
C12:0	0.23 ± 0.01^b^	0.20 ± 0.01^ab^	0.19 ± 0.01^a^
C13:0	0.93 ± 0.002	0.93 ± 0.001	0.93 ± 0.001
C14:0	1.50 ± 0.09	1.25 ± 0.03	1.34 ± 0.06
C14:1	0.07 ± 0.01^a^	0.08 ± 0.003^ab^	0.09 ± 0.01^b^
C15:0	0.30 ± 0.02	0.35 ± 0.02	0.31 ± 0.02
C15:1	0.14 ± 0.01^a^	0.15 ± 0.01^ab^	0.17 ± 0.01^b^
C16:0	10.34 ± 0.34	10.54 ± 0.10	10.40 ± 0.10
C16:1	0.95 ± 0.08	1.20 ± 0.16	0.91 ± 0.04
C17:0	2.40 ± 0.13	2.33 ± 0.14	2.15 ± 0.12
C17:1	0.55 ± 0.03	0.47 ± 0.05	0.52 ± 0.04
C18:0	10.56 ± 0.38	10.74 ± 0.29	10.13 ± 0.24
C18:1 T	1.40 ± 0.13	1.33 ± 0.14	1.18 ± 0.07
C18:1C	18.52 ± 0.31	18.54 ± 0.19	18.71 ± 0.14
C18:2C	4.29 ± 0.15^b^	4.43 ± 0.38^ab^	3.36 ± 0.18^a^
C18:3 (*n*-6)	0.07 ± 0.01	0.08 ± 0.01	0.06 ± 0.01
C18:3 (*n*-3)	0.40 ± 0.02^b^	0.34 ± 0.02^ab^	0.28 ± 0.01^a^
C20:3 (*n*-6)	0.17 ± 0.004	0.16 ± 0.01	0.18 ± 0.004
C22:1 (*n*-9)	0.05 ± 0.02	0.08 ± 0.02	0.06 ± 0.02
C20:4 (*n*-6)	3.19 ± 0.15^a^	3.46 ± 0.13^ab^	3.94 ± 0.09^b^
C24:0	0.41 ± 0.02	0.43 ± 0.01	0.40 ± 0.01
C22:6 (*n*-3)	0.14 ± 0.01	0.15 ± 0.01	0.13 ± 0.01
∑SFA^2^	26.73 ± 0.71	29.86 ± 0.37	25.93 ± 0.12
∑MUFA^3^	21.58 ± 0.44	21.68 ± 0.29	21.51 ± 0.09
∑PUFA^4^	8.25 ± 0.23	8.62 ± 0.29	7.95 ± 0.24
∑PUFA/∑SFA	0.31 ± 0.01	0.32 ± 0.01	0.31 ± 0.01
∑*n*-6PUFA	3.49 ± 0.15^a^	3.77 ± 0.12^ab^	4.24 ± 0.10^b^
∑*n*-3PUFA	0.54 ± 0.03^b^	0.49 ± 0.02^ab^	0.41 ± 0.01^a^
∑*n*-6/∑*n*-3	6.57 ± 0.48^a^	7.79 ± 0.52^ab^	10.36 ± 0.28^b^

^1^ Values are means of six replicates per treatment. Fatty acid content is based on freeze-dried condition. Group C = controls, Group MM = melatonin implanted in May group, Group MJ = melatonin implanted in June

^2^ SFA = saturated fatty acid

^3^ MUFA = monounsaturated fatty acid

^4^ PUFA = polyunsaturated fatty acid

^a, b^ Mean in the same row with different superscripts are significantly different (*P* < 0.05)

### Tissue levels of melatonin and prolactin

Melatonin concentration of lungs was highest (*P* < 0.05) in control goats (Group C) and this group had a lower (*P* < 0.05) mean prolactin concentration in lung tissue than the melatonin-implanted goats (Groups MM and MJ) (Table 5). Otherwise, there was little effect of the melatonin treatments on tissue levels of melatonin or prolactin in these goats (Table 5).

**Table 5:** Effects of melatonin implantation on melatonin and prolactin content of muscle and viscera in Inner Mongolian cashmere goats^1^

Items		*Longissimus dorsi*	*Biceps femoris*	*Gluteus*	Kidney	Heart	Spleen	Lung	Liver
Melatonin, pg/mg	Group C	2.74 ± 0.70	2.56 ± 0.67	2.19 ± 0.37	1.99 ± 0.18	2.43 ± 0.50	3.45 ± 0.40	6.23 ± 0.26	2.44 ± 0.70
	Group MM	2.65 ± 0.17	2.61 ± 0.99	1.96 ± 0.40	1.42 ± 0.26	2.19 ± 0.53	3.65 ± 0.64	4.16 ± 0.35	3.22 ± 0.80
	Group MJ	2.56 ± 0.30	2.70 ± 0.63	1.93 ± 0.46	1.84 ± 0.65	1.64 ± 0.23	3.59 ± 0.40	4.14 ± 0.36	4.08 ± 0.82
Prolactin, ng/mg	Group C	0.89 ± 0.09	1.10 ± 0.18	0.90 ± 0.08	1.18 ± 0.12	0.77 ± 0.13	0.61 ± 0.07	0.31 ± 0.03	0.86 ± 0.14
	Group MM	1.01 ± 0.27	1.09 ± 0.27	0.89 ± 0.08	0.86 ± 0.06	0.84 ± 0.10	0.51 ± 0.06	0.61 ± 0.06	0.74 ± 0.18
	Group MJ	0.94 ± 0.08	0.93 ± 0.06	0.90 ± 0.13	1.16 ± 0.20	0.92 ± 0.11	0.57 ± 0.07	0.55 ± 0.06	0.76 ± 0.11

^1^ Values are means of six replicates per treatment. Group C = controls, Group MM = melatonin implanted in May group, Group MJ = melatonin implanted in June

^a, b^ Mean in the same column of each item with different superscripts are significantly different (*P* < 0.05)

## Discussion

The melatonin implantation mode used in this test has been proved that have a significant effect on the melatonin concentration in plasma on the basis of the prior works repeatedly [7, 22], however there was no effect of melatonin treatment of these cashmere goats on carcass characteristics and the only notable effects on meat quality were slight reductions in protein content and indices of cooking quality, and some increase in shear force (toughness), which were detectable at 1 month after implantation but had largely disappeared 1 month later. This finding contrasts with a masterate study of pigs [15] in which melatonin appeared to produce dose-related effects on carcass characteristics, however the dosages used in the pigs were vastly higher than that used here in the goats.

There are very few studies on the effects of melatonin treatments on meat quality and none have been reported in the scientific literature for cashmere goats. It is noteworthy that the pH values of meat from cashmere goats in the present study (range 5.83 to 6.54) are indicative of good carcass quality [23] and fall within the range reported in a masterate study of 1.5-year-old Inner Mongolian cashmere nanny goats (5.9 to 6.5) [24]. Likewise, muscle color which depends on the pigment (myoglobin and hemoglobin) content, chemical state and light scattering properties of meat [25] and is critical for maintaining an attractive appearance of the flesh, was not affected by melatonin treatment of the goats in the present study. Other important attributes of meat include tenderness [26] which manifests as lowered shear force [27], and cooking performance which is related to its water holding capacity [28–30]. Although there were minor changes in some of these properties of meat from the melatonin-treated cashmere goats, they were inconsistent or transitory. In general, the relatively low fat content of goat flesh makes it attractive to consumers seeking a lean red meat [31, 32].

There is some evidence that treatment of animals with melatonin may alter their metabolism. For instance melatonin treatment lowered intraabdominal adiposity of rats [33] and affected the relationship between blood glucose levels and their regulatory signaling pathways [33–36]. It cannot be determined whether such effects occurred in the present study but the absence of major changes in carcass composition tend to rule out any significant events of this nature. The apparently normal occurrence of essential and non-essential amino acids in the muscles of these goats also showed no negative effects of the melatonin treatment and provided a favorable comparison with international standards for ideal protein composition from the World Health Organization (WHO) and the Food and Agriculture Organization of the United Nations (FAO) [37] (Table 6). A similar picture applies to the fatty acid composition of the muscles where any effects of melatonin treatment were relatively minor and had largely disappeared at 2 months.

**Table 6:** Essential amino acids and ideal protein ratio in Inner Mongolian cashmere goats^a^

Amino acids	FAO/WHO ideal protein, mg/g	*Longissimus dorsi*	
		AA, mg/g^b^	AAS, %^c^
Thr	40	36.17	90.43
Val	50	41.70	83.40
Met + Cys	35	27.83	79.51
Ile	40	40.13	100.33
Leu	70	68.83	98.33
Phe + Tyr	60	73.73	122.88
Lys	55	80.73	146.78

^a^Amino acid content is based on freeze-dried condition. The data are the average value of the three groups of Inner Mongolian cashmere goats

^b^Values of AA are means of all samples including the three treatment groups

^c^*AAS* Amino acid score, represents the ratio of amino acid content in the *Longissimus dorsi* muscle to the ideal protein model

Melatonin is an endogenous substance that is rapidly cleared from the body; with half-lives of 17.8, 19.8, 18.6 and 34.2 min in sheep, rats, dogs, and monkeys, respectively [38, 39]. It is mostly (70–75%) metabolized to 6-hydroxy melatonin sulfate in the liver, of which about 80% is excreted in the urine and 20% in feces. There was no evidence that the exogenous melatonin had accumulated in body tissues of the goats, although this may be attributable to the low dose that was used here (2 mg/kg). Although there are established links between melatonin and the regulation of prolactin secretion [40, 41], the only connection detected here was an elevation of prolactin content in lungs of melatonin-treated goats. The significance of this observation is not clear.

A general, and notable, feature of these results is that many of the effects that were recorded after 1 month of melatonin implantation were not evident at 2 months of implantation. This indicates that the exogenous supply of melatonin may have caused some physiological changes but these were overcome by subsequent compensatory adjustments or by loss of responsiveness to the stimulus. After the sustained release period, the effects of exogenous melatonin will not sustain.

## Conclusion

Implantation of cashmere goats with a slow release formulation of melatonin (lasting 1 to 2 months) in order to boost production of quality cashmere fiber does not impact unfavorably on production of meat nor affect its composition and quality. Also there is no significant residue, or accumulation, of the administered melatonin in the various organs and tissues. This study has provided an empirical basis for the use of melatonin in cashmere production systems.

## Data Availability

All data generated or analyzed during this study are included in this published article.

## Abbreviation

PUFA: Polyunsaturated fatty acid
